# Exploring Microbial Dark Matter for the Discovery of Novel Natural Products: Characteristics, Abundance Challenges and Methods

**DOI:** 10.4014/jmb.2407.07064

**Published:** 2024-11-18

**Authors:** Abdullah R Alanzi

**Affiliations:** Department of Pharmacognosy, College of Pharmacy, King Saud University, Riyadh 11451, Saudi Arabia

**Keywords:** Microbial, dark matter, natural products, isolates, genetic, phylogenetic, single-cell genomics, metagenomics, and culturomics

## Abstract

The objective of this review is to investigate microbial dark matter (MDM) with a focus on its potential for discovering novel natural products (NPs). This first part will examine the characteristics and abundance of these previously unexplored microbial communities, as well as the challenges faced in identifying and harnessing their unique biochemical properties and novel methods in this field. MDMs are thought to hold great potential for the discovery of novel NPs, which could have significant applications in medicine, agriculture, and industry. In recent years, there has been a growing interest in exploring MDM to unlock its potential. In fact, developments in genome-sequencing technologies and sophisticated phylogenetic procedures and metagenomic techniques have contributed to drastically make important changes in our sights on the diversity of microbial life, including the very outline of the tree of life. This has led to the development of novel technologies and methodologies for studying these elusive microorganisms, such as single-cell genomics, metagenomics, and culturomics. These approaches enable researchers to isolate and analyze individual microbial cells, as well as entire communities, providing insights into their genetic and metabolic potential. By delving into the MDM, scientists hope to uncover new compounds and biotechnological advancements that could have far-reaching impacts on various fields.

## Introduction

In the vast tapestry of microbial life, a significant portion remains shrouded in mystery, escaping traditional cultivation methods and defying characterization – a realm aptly termed as microbial dark matter (MDM) [1]. This enigmatic domain encompasses a myriad of uncultured microorganisms that hold immense potential for harboring novel NPs with transformative applications in medicine, agriculture, and biotechnology. As we embark on a journey to explore MDM, the challenges and opportunities within this uncharted territory become increasingly apparent. In fact, MDM research has gained momentum in recent years, driven by technological advancements and a growing recognition of its profound implications [2]. The microbial world, traditionally explored through culture-based methods, represents only a fraction of the diverse microorganisms populating our ecosystems. Traditional methods of investigating MDM involve analyzing the diversity of uncultivated microorganisms from various environments through culture-based approaches, environmental sampling, and then sequencing. One of the strengths of these methods is that they could give a broad sense of microbial diversity and community structure [3]. Traditional culture-based techniques have both advantages and disadvantages for researching MDM. On the positive side, culturing organisms allows for direct observation and experimentation that can provide insights into microbial physiology, biochemistry, and ecology [4]. Isolating living microbes in pure culture is necessary for detailed laboratory investigation. However, culture-based methods are also very limiting as the vast majority of microbes, estimated at over 99%, cannot be cultured using standard techniques [5]. This means traditional approaches only scratch the surface of microbial diversity. They provide an extremely narrow view of the actual complexity and functions within communities. Modern culture-independent methods using DNA sequencing and metagenomics have transformed our ability to study uncultured MDM at a much higher resolution. While losing the ability to grow isolates, these new techniques allow researchers to comprehensively analyze entire microbial consortia directly from environmental samples. [6]. Thus, traditional methods, although greatly contributing to MDM research, are also burdened with specific challenges that obviously call for the development of more innovative approaches. Interestingly, the limitations of conventional techniques have led scientists to delve into the largely unexplored genetic and metabolic landscape of uncultured microbes. Perceptibly, metagenomics, single-cell genomics, and cultivation-independent methods have emerged as powerful tools, enabling researchers to peer into the genetic material of microbial dark matter without the constraints of traditional cultivation. Metagenomic approaches provide a collective snapshot of the genomic content of entire microbial communities, offering insights into the potential functions and interactions of uncultured microorganisms. Single-cell genomics, on the other hand, allows for the isolation and genomic analysis of individual cells, offering a finer resolution in deciphering the genetic diversity within MDM [7].

The impressive track record of ground-breaking discoveries from uncultivated microorganisms highlights the significance of investigating MDM for the discovery of NPs. These discoveries, which have transformed medicine, include immunosuppressants, antibiotics, and anticancer drugs [8]. The possibility of discovering new molecules with medicinal potential exists within the enormous store of unexplored genomic and metabolic diversity found in MDM. It is imperative that we take into account the difficulties involved in researching MDM as we set out on this investigation [9]. Difficult challenges include limited cultivability, lack of reference genomes, and complexity of functional annotation. But with teamwork, interdisciplinary thinking, and the incorporation of state-of-the-art technologies, science is finally going to be able to reveal the mysteries of MDM [10].

In this journey of discovery, this exploration of MDM not only deepens our understanding of the microbial world but also holds the potential to address global challenges through the identification of novel NPs. This literature of review sets the stage for a comprehensive exploration of the challenges, methodologies, and future prospects in MDM research for the discovery of novel NPs.

## Overview of MDM and the Challenges in the Discovery of Novel NPs from This Group

MDM encompasses the vast spectrum of microbial organisms, predominantly bacteria and archaea, that elude cultivation in laboratory settings due to a lack of knowledge or the inability to replicate their specific growth conditions. Diverging from the concept of dark matter in physics and cosmology, MDM earns its nomenclature owing to the intricate challenge it poses in terms of study, arising from its resistance to conventional culturing methods. The magnitude of MDM remains challenging to estimate precisely, with a widely accepted gross approximation suggesting that as little as one percent of microbial species within a given ecological niche can be successfully cultured. In recent years, a concerted effort has been directed towards unraveling the mysteries of MDM. This pursuit involves the recovery of genome DNA sequences from environmental samples using innovative, culture-independent methods such as single-cell genomics and metagenomics. These groundbreaking studies have not only provided insights into the evolutionary history and metabolic pathways of the sequenced genomes but have also furnished essential knowledge necessary for advancing the cultivation of MDM lineages. Through these endeavors, scientists are gradually peeling back the layers of microbial complexity, shedding light on a previously obscured realm and paving the way for unprecedented discoveries in microbiology and beyond.

**Fig. 1:**
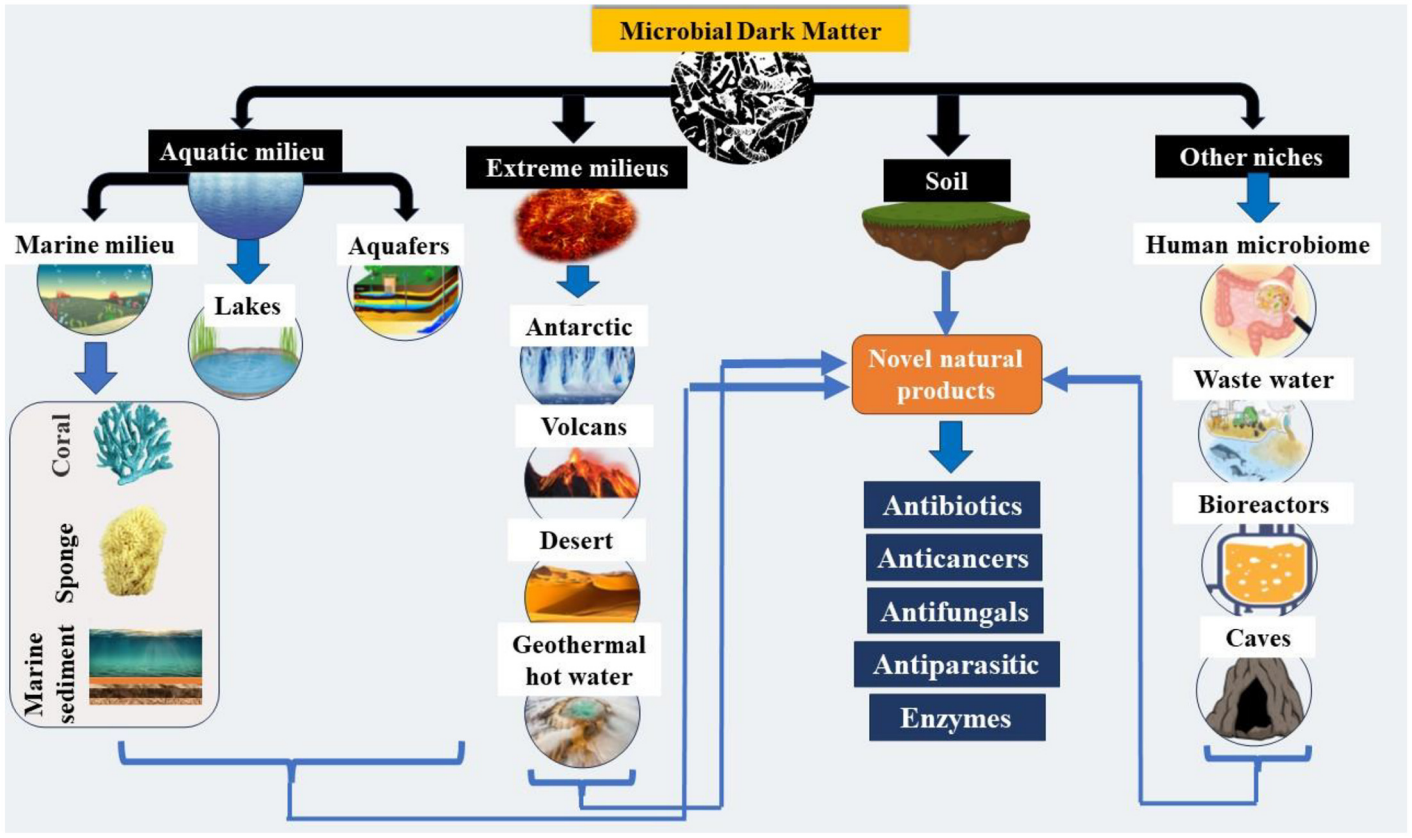
Microbial dark matter, residing in diverse ecological niches, constitutes a fascinating and enigmatic realm within the microbial world.

Diverse microbiomes have been found in various environments such as marine, freshwater, soil, and the human gut, making them potential sources of metagenomic DNA; this is represented in Fig. 1. Evidently, bioactive chemicals that are active in live cells, tissues, and organisms are known as bioactive compounds. Due to the fact that these substances are not directly connected to the growth and development of the producing organism, they are also referred to as Secondary metabolites (SMs) [11]. A particular organism or a related group of species create particular categories of bioactive chemicals. Additionally, bioactive compounds have the ability to function as antioxidants and effectively combat free radicals. Many human diseases, such as cancer and microbial infectious diseases, which use oxygen free radicals in their pathogenesis, can be avoided by eliminating reactive oxygen species [12].

SMs and NPs have been the subject of extensive study, with a particular emphasis on soil microbiomes. Actinobacteria, a prevalent phylum within soil communities, have garnered attention for their remarkable capacity to produce a diverse array of SMs [13]. However, with the advent of high-throughput sequencing, it has become abundantly clear that ecosystems traditionally considered understudied, along with the MDM—representing the vast majority of unculturable microorganisms-harbor tremendous biosynthetic potential, holding promise for future drug discovery endeavors [14]. Despite this revelation, our understanding of how this potential is distributed and the factors influencing it remains limited. Unraveling the intricacies of these unexplored biosynthetic landscapes is crucial for unlocking novel therapeutic compounds and advancing our comprehension of microbial communities beyond traditionally studied environments [15].

The revelation that a significant proportion of NPs is governed by gene clusters, accessible through bacterial genome sequence data or communal bacterial genome sequences, has recently sparked intensified research in this field. Recent studies indicate that numerous adept producers of small compounds remain uncultivable in laboratory settings, underscoring the notion that the biosynthetic potential of bacteria is still largely unexplored [16]. Intriguingly, even among bacteria successfully cultured in labs, the number of biosynthetic gene clusters exceeds the count of experimentally extracted natural compounds [17]. The advent of high-throughput sequencing tools in contemporary research provides a powerful means to identify "biosynthetic dark matter," previously undiscovered components of bacterial biosynthesis, unraveling hidden potential within microbial communities that has eluded detection until now.

## Success Stories Developed through MDM Research

MDM research has catalyzed several remarkable discoveries that highlight the vast potential of this emerging field. One of the most significant findings is the identification of the candidate phylum *Lokiarchaeota*, named after a deep-sea hydrothermal vent in the North Atlantic where it was first discovered [18]. Through innovative MDM approaches, scientists were able to reconstruct nearly complete genomes of this previously unknown archaeal lineage. This remarkable achievement provided insights into its metabolic capabilities, illustrating that *Lokiarchaeota* may possess unique enzymatic functions that allow it to thrive in extreme environments [19]. Furthermore, the research has revealed evolutionary connections between *Lokiarchaeota* and eukaryotes, sparking discussions about the origins of complex life forms. Another noteworthy example of MDM research is the characterization of *Thorarchaeota*, another candidate phylum uncovered in deep subsurface environments [20]. Utilizing advanced metagenomic techniques, researchers have elucidated the metabolic versatility of *Thorarchaeota*. This lineage appears to play crucial roles in global biogeochemical cycles, particularly in the cycling of carbon and nitrogen. By understanding how *Thorarchaeota* contributes to these processes, scientists can better predict the impacts of environmental changes on ecosystem dynamics [21]. These discoveries underscore the immense power of MDM research, unveiling a hidden world of microbial diversity that has previously evaded cultivation and characterization. The insights gained from studying these uncultivated microorganisms not only deepen our understanding of microbial evolution and ecology but also pave the way for groundbreaking advancements in various fields, including biotechnology, environmental science, and health [19]. While MDM continues to be investigated by researchers, other such hidden treasures are likely to emerge that could bring about a complete transformation in our way of comprehending the microbial world and its vital contributions toward Earth's ecosystems.

## Characteristics and Abundance of MDM

Beyond cosmic dark matter, another enigmatic realm of darkness envelops our daily lives – a domain composed of bacteria and microorganisms existing outside the human body, awaiting the scrutiny of genetic sequencing and laboratory cultivation to unveil their defining characteristics. In stark contrast to the extensively cataloged bacteria residing within our bodies, this MDM remains largely uncharted. It permeates the vast majority of ecosystems on our planet, housing an immense diversity of microorganisms yet to be fully explored and understood [22].

Since the 1960s, microorganisms, including fungi, eubacteria, and archaea, thriving in harsh terrestrial environments have been subject to the extraction and structural description of their bioactive SMs. This exploration extends to a diverse array of naturally occurring products derived from terrestrial extremophiles, a term the authors eschew. These microorganisms are ubiquitously present in terrestrial landscapes, from land and rivers to salterns, but they are notably absent in aquatic environments deeper than seashores. This study categorizes extremophiles based on their isolation and bioactivity, encompassing metallotolerant, radioresistant, unclassifiable microorganisms, alkaliphiles, halophiles, xerophiles, thermophiles, psychrophiles, acidophiles, and endophytes. The research not only delineates these extremophiles but also provides insight into the medication evaluation process of several SMs derived from them. These metabolites are currently undergoing scrutiny in (pre)clinical trials, marking a significant step forward in understanding their therapeutic potential [23].[Fig F2]

## Challenges associated with Limited Cultivability and Cultivation-Dependent Bias

**Fig. 3:**
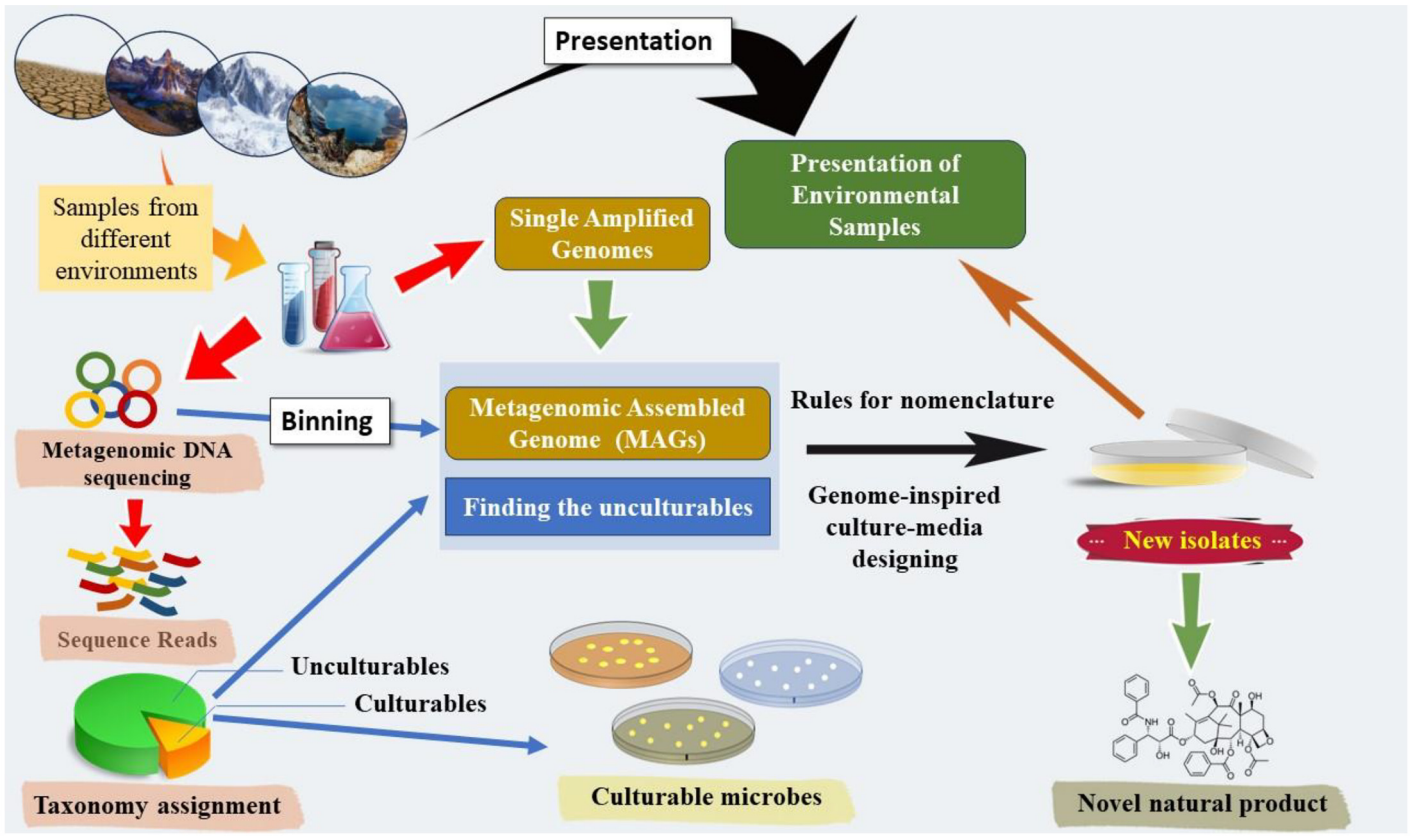
The outlined schematic encapsulates the workflow for designing media by leveraging genomic information derived from metagenomic assembled genomes (MAGs). This figure was redesigned from the figure of Sood *et al*. [84].

With the advent of culture-independent DNA sequencing approaches, such as metagenomics, researchers have been able to circumvent the limitations imposed by traditional culturing methods. Next-generation sequencing (NGS) technologies have played a pivotal role in revolutionizing the field, enabling the direct sequencing of genetic material from environmental samples without the need for cultivation (Fig. 3). This breakthrough has significantly expanded our understanding of microbial diversity by uncovering the genomes of previously unculturable microorganisms. In fact, annotation pipelines, which automatically identify and label the functional elements of a genome, have further propelled the analysis of these vast datasets. The amalgamation of culture-independent sequencing and advanced annotation tools has not only enhanced our awareness of elusive microorganisms but has also paved the way for exploring the functional potential encoded within their genomes [24].

Despite these advancements, a substantial portion of the functional genomic content remains enigmatic. The functions associated with specific genes or genetic elements are often unknown, presenting a challenge in translating genomic information into actionable biological knowledge [25]. In this section, we will delve into studies that sought to quantify and characterize the Functional Dark Matter (FDM) of microbial genomes. The term "Functional Dark Matter" refers to the portion of genomic content whose functions have yet to be elucidated. In this respect, researchers have employed diverse strategies to unravel the mysteries of FDM, ranging from computational approaches to experimental validations [26]. Quantitative studies aim to assess the abundance and distribution of FDM across different microbial communities, shedding light on the prevalence and potential significance of uncharacterized genetic elements. Concurrently, efforts have been made to exploit the functional richness encapsulated within the FDM, with the goal of uncovering novel biological insights with direct biotechnological implications [27]. By exploring the untapped functional potential encoded in the genomes of microorganisms, these studies contribute not only to our fundamental understanding of microbial ecology and evolution but also hold promise for the discovery of novel enzymes, pathways, and bioactive compounds with applications in various biotechnological sectors. In the subsequent sections, we will examine specific methodologies employed in FDM research, the challenges encountered in deciphering its functional landscape, and the potential applications of this knowledge in biotechnology.

Microorganisms can exist either as independent entities or form symbiotic associations with multicellular hosts or other unicellular organisms across diverse ecological habitats, ranging from the depths of seas to arid deserts and the internal environments of multicellular organisms, including certain symbiotic or parasitic systems [28]. The microbial landscape is extensive, with approximately 4 × 10^6^ distinct microbial taxa and 10^9^ cell per ton and per gram of soil samples, respectively. The microbial diversity within soil is estimated to encompass between 3000 and 11,000 genomes per gram [29]. Despite this vast microbial richness, cultivation techniques grant access to less than 1% of the microorganisms in ecosystems, leaving more than 99% unstudied. This limitation restricts our understanding of the morphology, metabolism, genetics, and population ecology of these uncultured microorganisms [30]. The cultivation and study of these microorganisms under natural growth conditions present ongoing challenges [31]. Consequently, the identification of NPs from these uncultured or uncharacterized microbes has been a persistent challenge, impeding comprehensive exploration and exploitation of their biochemical potential for an extended period.

## Specific Technological Advancements

Recent technological advancements have transformed our ability to explore MDM, unveiling a vast repository of novel NPs. High-throughput metagenomic sequencing now enables researchers to analyze the genetic diversity and functional potential of entire microbial communities directly from environmental samples, bypassing the need for cultivation. As metagenomic datasets have grown in size and complexity, advances in bioinformatics tools have facilitated large-scale data analysis. This allows researchers to screen metagenomic sequences for biosynthetic gene clusters involved in producing SMs, providing insights into the diverse chemical capabilities of uncultivated microbes.

In addition, single-cell genomics and high-resolution mass spectrometry have enhanced the characterization of microbial communities at the single-cell level, overcoming previous barriers to isolating and studying uncultured species. Collectively, these innovations have significantly broadened our understanding of MDM, revealing its abundance and potential for natural product biosynthesis. Continued technological developments are pushing the boundaries of this exploration, promising new discoveries with applications in drug development, sustainable agriculture, and biotechnology. As we learn to more fully harness this vast source of biodiversity and chemical diversity, the future of NPs research holds immense promise.

### Latest Technologies such as Single-Cell Genomics, Metagenomics, and Cultivation-Independent Methods, Being Employed to Study MDM

Even after 130 years of tireless cultivation efforts, countless microorganisms, including entire candidate phyla of bacteria and archaea, remain elusive to traditional approaches. Delving into the secrets of these enigmatic microbes presents a formidable challenge, but one with profound implications for understanding both extreme environments and the vast, uncultivated majority of life on Earth. The mysteries surrounding uncultivated candidate phyla, abundant in low-energy ecosystems like deep-sea vents and subterranean ice cores, represent a major frontier in microbiology. Unraveling their metabolic quirks, ecological roles, and potential applications in biotechnology requires innovative approaches, promising to expand our understanding of lifés intricate tapestry [32]. While contemporary culture-independent techniques have demonstrated considerable efficacy in unveiling the structural intricacies of microbial communities and identifying biosynthetic gene clusters within microbial genomes [33], it remains imperative to isolate pure microbial strains. This conventional approach is essential to comprehensively evaluate the characteristics and potential applications of the SMs produced by these microorganisms [85]. Despite the advancements in culture-independent methods, the isolation of pure strains remains a fundamental step in understanding the complete spectrum of microbial activities and unlocking the full potential of their SMs [34]. Rapid progress on this issue has been made possible over the past ten years by parallel developments in DNA amplification, DNA sequencing, and computation, especially in the areas of metagenomics and single-cell genomics [86]. While both methodologies have their drawbacks, metagenomics and single-cell genomics work especially well together. The first significant genomic data for a number of candidate phyla, including putative acidophiles (*Parvarchaeota*), halophiles (*Nanohaloarchaeota*), thermophiles (*Acetothermia*, *Aigarchaeota*, *Atribacteria*, *Calescamantes*, *Korarchaeota*, and *Fervidibacteria*), and piezophiles (*Gracilibacteria*), have been uncovered by studies focused on extreme environments [32].

In a recent study, Andre and his coworkers, described uncultured, or resistant, microorganisms from an Antarctic soil sample utilizing comparatively straightforward techniques: slow-growing bacterium selection, oligotrophic medium, prolonged incubation times, and stereomicroscopy observation. Hence, several uncommon bacteria from seldom isolated or recently described taxa, including *Lapillicoccus*, *Flavitalea*, *Quadrisphaera*, *Motilibacter*, and *Polymorphobacter*, were successfully isolated by these researchers. Furthermore, two separate isolates from the class *Thermoleophilia* were obtained, which, despite being common in Antarctic soils (as determined by metagenomics), had never been reported to be isolated from such samples. These isolates showed 16S rRNA sequence similarity ranging from 92.08 to 94.46% with any other known cultured species. Our findings suggest that, even when working with samples from harsh settings, straightforward techniques can still be effective in growing resistant microbes [35].

**Single-cell genomics: breaking down microbial diversity at the single-cell level.** The majority of microbial diversity on our planet is attributed to uncultured microorganisms. Despite significant advancements in environmental sequencing and single-cell genomics, the in-depth exploration of bacterial metabolism and the discovery of novel bioproducts necessitate the cultivation of microbes in a laboratory setting. While modern molecular techniques provide valuable insights into the genetic makeup and diversity of microbial communities, the cultivation of microorganisms remains a fundamental approach for a more comprehensive understanding of their metabolic capabilities and the potential applications of their bioactive compounds. Laboratory culturing not only allows for the characterization of microbial physiology but also facilitates the screening and identification of valuable bioproducts with diverse industrial and medical applications [87]. In this context, the integration of both cultivation-based and culture-independent approaches is essential for unlocking the full spectrum of microbial diversity and harnessing its biotechnological potential. Herés a refined and expanded version of the paragraph:

When it comes to the precise isolation and delivery of individual cells, micromanipulation techniques are essential, providing unparalleled visual control with the aid of high-resolution microscopes. Within this realm, two prominent methods stand out: the traditional micropipette and the cutting-edge optical tweezers. The micropipette, a familiar tool in any laboratory, seamlessly integrates with inverted microscopes and established liquid handling systems. While its user-friendly nature makes it a natural choice for small-scale operations, its labor-intensive process and limited throughput (typically averaging around 50 cells per hour per person, as highlighted by Picelli in 2016) render it less suitable for larger tasks [36]. On the other hand, optical tweezers utilize light itself to delicately manipulate cells with minimal physical contact [37]. This finesse, however, comes with a higher learning curve and potentially greater initial investment. Ultimately, the selection between these micromanipulation marvels depends on the desired scale and complexity of your single-cell endeavors [38].

Single cell genomics (SCG) has quickly evolved over the last ten years from science fantasy to a useful new tool for biologists. The capability of this technology to extract information-rich genomic blueprints from individual cells, the most basic units of biological organization, is what gives it its strength. This is especially important for protists, bacteria, and archaea, since individual cells make up entire organisms [88]. Though only a small portion of the microbial diversity has been found and investigated, such unicellular organisms make up the vast bulk of the biological diversity and biomass of our planet [39]. In contrast to the comprehensive approach employed in metagenomics, single-cell genomics offers the ability to investigate genomes on a cellular level, allowing for a profound exploration of organisms at their most fundamental biological unit [40]. This technique involves the isolation of individual cells from a complex mixture, followed by the subsequent steps of cell lysis and the amplification of genomic DNA. Various methods, including fluorescence-activated cell sorting (FACS) or optofluidics [41], are utilized for the isolation of single cells, each presenting distinct advantages depending on the specific application [42]. In recent years, random encapsulation approaches, particularly utilizing flow cytometry and microfluidic devices, have gained widespread prominence. Flow cytometry and FACS, known for their significantly higher throughput, have proven to be effective platforms for conducting single-cell analysis in microbial cells [43]. In fact, post-isolation, the genomic DNA from individual cells, often characterized by its inefficiency [2], undergoes amplification using multiple displacement amplification (MDA) [44] or alternative genome amplification approaches. This amplification process elevates the DNA content from femtogram levels in an individual cell (approximately 1 fg per Mbp) to the nanogram- to microgram-levels necessary for subsequent sequencing. The resultant single amplified genomes (SAGs) are then subjected to initial screening through PCR amplification and sequencing of SSU rRNA genes, facilitating the identification of those associated with candidate phyla or other taxa of interest. Actually, SAGs of particular interest subsequently undergo shotgun sequencing, assembly, and comprehensive analysis [89]. The assessment of the number of single-copy conserved markers (SCMs) in the assembly provides an estimate of the coverage of the target organism's genome by a given SAG [42]. This intricate process enables a detailed exploration of microbial genomics at the single-cell level, offering valuable insights into the diversity and characteristics of microbial communities. Single-cell genomics offers a way to skip the culturing process and directly study environmental microbes at the individual cell level. This technique has proven effective in investigating little-known archaeal and bacterial candidate phyla, often referred to as MDM. This approach is based on outlining the single-cell genomics workflow, covering sample preparation and preservation, high-throughput fluorescence-activated cell sorting, cell lysis, and amplification of environmental samples. Additionally, the use of phylogenetic screening based on 16S rRNA genes and sequencing approach are required for this technique [90]. In a recent study, Wiegand *et al*. utilize the microfluidic single-cell dispenser system for the first time to isolate microorganisms from a complex environmental sample. The sample source was a wastewater treatment plant that has previously been shown to be rich in Patescibacteria/CPR bacteria. Through this study we were able to increase the knowledge on the genomic diversity of this understudied clade. We could demonstrate that some of the major drawbacks of FACS, e.g., the lack of single-cell visualization (and therefore proof of consistent cell separation) and suspected pressure induced cell stress that may affect cell yield and purity during FACS sorting can be positively addressed, enabling a variety of alternative downstream applications for single cell sorting [45].

Briefly, thus technique involves isolating individual cells from environmental samples and amplifying their DNA for sequencing [45]. This approach has been successfully used to sequence genomes of previously uncultured microorganisms, providing valuable insights into their biology and ecology [46]. By using this powerful tool, researchers can unlock the secrets of the MDM and gain a deeper appreciation for the complexity and diversity of life on Earth.

**Metagenomics: unlocking the genomic potential of entire microbial communities.** Metagenomics involves examining the combined genetic material of microorganisms within a community, offering valuable insights into the genomic landscape of uncultured environmental microbes [7]. Metagenome sequencing typically employs a non-discriminatory shotgun sequencing approach, facilitating taxonomic assignment and organism quantification down to the species level. Additionally, this method enables functional annotation of identified genes. A pivotal phase in the analysis is metagenome sequence assembly, a process that involves stitching together individual DNA sequences [47]. Metagenome-assembled genomes (MAGs) are derived through the binning of assembled contigs sharing similar characteristics, followed by quality filtering [48]. Consequently, MAGs can represent the genomic profile of closely related microbial strains within the community. This strategy represents a groundbreaking approach in genomics, poised to unlock the vast genomic potential inherent in entire microbial communities. Unlike traditional genomics, which focuses on individual organisms cultured in a laboratory setting, metagenomics delves into the collective genetic material of diverse microorganisms coexisting in their natural environments. This innovative field enables scientists to explore the genetic diversity, functional capabilities, and interactions within complex microbial ecosystems. Imagine peering into a hidden world teeming with trillions of tiny inhabitants, each with its own unique genetic code [49]. Think of DNA as the blueprint of life, and a microbial community as a vibrant city bustling with different residents. Each microbe, from bacteria to archaea, carries its own DNA blueprint, holding secrets about its functions, adaptations, and potential benefits. Traditionally, studying these microbes meant isolating them in a lab, a slow and often unreliable process that missed the rich tapestry of interactions within the community.

The essence of metagenomics lies in its ability to capture a holistic snapshot of microbial communities, providing a comprehensive understanding of their genetic makeup. This approach is particularly crucial in environments where the majority of microorganisms elude cultivation in the lab. Metagenomics employs advanced DNA sequencing technologies to analyze and characterize the collective genetic content, or metagenome, of these communities, thereby uncovering hidden genetic treasures that may hold the keys to numerous biological processes and functions. Obviously, the applications of metagenomics are far-reaching, ranging from environmental studies and biotechnology to human health . By unraveling the genomic potential of microbial communities, researchers can identify novel genes, enzymes, and pathways with diverse functionalities. This wealth of genetic information opens avenues for the discovery of new biotechnological applications, the understanding of microbial community dynamics, and the development of innovative approaches in fields such as medicine, agriculture, and environmental management [91].

Metagenomics is still in its early stages, but its potential is vast. It's like opening a window into a previously unseen world, offering us a glimpse into the hidden forces that shape our planet, our health, and the very essence of life on Earth. As we delve deeper into the secrets of the metagenome, we unlock a treasure trove of knowledge and opportunity, waiting to be explored. The choice of an effective sampling strategy is emphasized as it directly influences the representation of microbial communities in the collected samples, impacting the accuracy and relevance of subsequent analyses [92].

In essence, metagenomics serves as a powerful tool for exploring the intricate tapestry of microbial life, particularly those belonging to MDM communities, providing insights into the roles microorganisms play in shaping their ecosystems and influencing broader biological processes. The unlocking of the genomic potential of entire microbial communities through metagenomics not only expands our knowledge of microbial diversity but also holds promise for transformative breakthroughs with significant implications across various scientific disciplines.

**Cultivation-independent methods: advancements in studying microbes in their natural environments.** For centuries, microbiologists have peered into the unseen world through the lens of cultivation. But for most microbes, this lens offered a distorted picture, capturing only a tiny fraction of the vast microbial diversity residing in their natural environments. Imagine a vibrant cityscape bustling with activity, but most residents are locked indoors. That's how it was with cultivation-based methods: only the readily cultivable microbes got studied, while the majority, adapted to complex ecosystems, remained hidden. Cultivation-independent methods are like tearing down the walls, offering us unfiltered access to the “entire” microbial community.

Cultivation-independent methods, such as metagenomics, metatranscriptomics, and metaproteomics, have allowed researchers to study microbial communities without the need for isolating and culturing individual microorganisms [50]. These methods involve the direct extraction and analysis of genetic material, RNA, or proteins from environmental samples, providing a more comprehensive view of the microbial diversity and functional potential in a given ecosystem [93]. Metagenomics, for example, involves the sequencing of DNA from an environmental sample, allowing for the identification of a wide range of microorganisms and their genetic capabilities. Metatranscriptomics focuses on the analysis of RNA to understand gene expression patterns within microbial communities, while metaproteomics involves the study of the proteins present in a given environment, providing insights into the metabolic activities of the microbial populations [94]. These cultivation-independent approaches have been instrumental in uncovering the previously unknown diversity and functional potential of microbes in various environments, including soil, water, and the human microbiome. They have also been crucial in understanding the roles of microbes in biogeochemical cycling, bioremediation, and human health [51].

The application of cultivation-independent methods has broad implications across various scientific disciplines. In environmental microbiology, these techniques contribute to our understanding of microbial ecology, nutrient cycling, and biogeochemical processes. In medicine, they aid in studying the human microbiome and its role in health and disease. Additionally, these methods have applications in biotechnology, agriculture, drug discovery and NPs production and other several industrial settings.

**Fig. 4:**
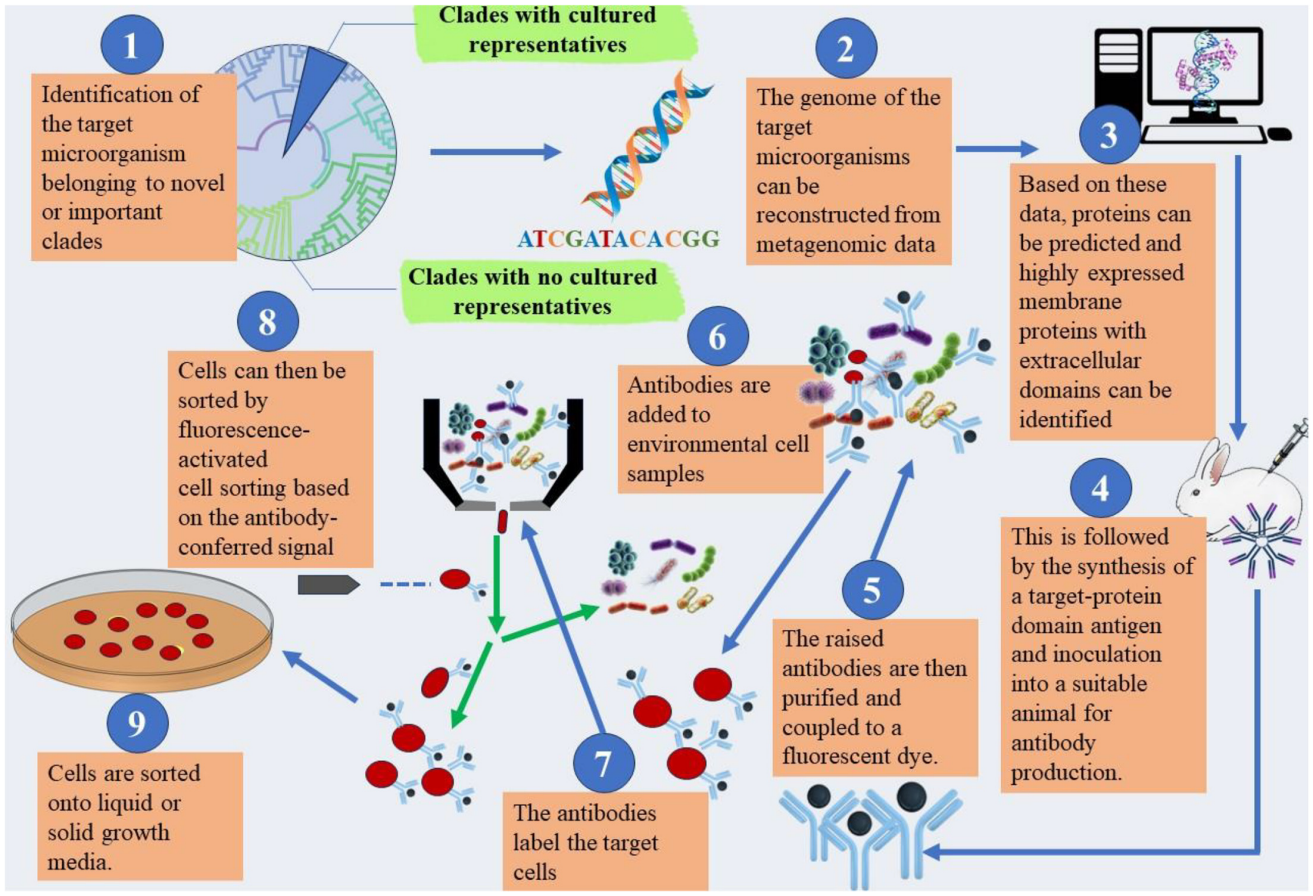
Harnessing Reverse Genomics for the targeted isolation and cultivation of novel Microorganisms from microbial dark matter.

**Other innovative techniques.** Traditionally, biologists unravel the mysteries of genes by observing their effects on outward traits. But reverse genomics flips the script, taking a targeted approach. Imagine having a specific gene in mind, perhaps one identified through a genetic study linked to a disease. Reverse genomics allows researchers to directly manipulate this gene and observe the resulting changes [52]. This can involve silencing the gene to understand its function, introducing altered versions to see how they influence the organism, or even creating entirely new organisms lacking the gene. By studying these "gene knockouts" or genetic modifications, researchers gain valuable insights into gene function, disease mechanisms, and potential therapeutic targets. It's like conducting a controlled experiment within the living organism, offering a powerful tool for unlocking the secrets of our genetic code (Fig. 4) [95].

Traditionally, sorting cells relied on fluorescent labels or antibodies, limiting studies to populations expressing specific markers. Raman-activated cell sorting (RACS) shatters this barrier, offering label-free, real-time identification and isolation based on a cell's inherent biochemical fingerprint. This powerful technique leverages Raman spectroscopy, which analyzes the vibrational signatures of molecules, providing a unique "molecular ID" for each cell [96]. Imagine shining a light on a diverse crowd, not to see their clothes, but to instantly recognize them by their unique biomolecular makeup. RACS does just that, capturing the subtle vibrations of cellular components like lipids, proteins, and DNA. Advanced algorithms then analyze these spectral fingerprints, enabling researchers to sort cells based on specific metabolic states, developmental stages, or even disease signatures [53]. The advantages are substantial: RACS preserves cell viability, eliminates labeling biases, and opens doors to studying previously hidden cell populations lacking suitable markers. Moreover, miniaturized microfluidic RACS platforms are emerging [54], promising high-throughput sorting for large-scale studies. This label-free revolution is reshaping cell biology, enabling groundbreaking research in cancer stem cell isolation, rare cell analysis, and personalized medicine. As RACS technology continues to evolve, we can expect even deeper insights into the remarkable diversity and dynamic landscapes of the cellular world.

Live-FISH, a groundbreaking technique that lets scientists visualize specific genes within living cells. Using fluorescently labeled DNA probes, researchers can literally tag and track the activity of individual genes in real-time, watching them flicker on and off within the cellular metropolis. From unravelling the secrets of embryonic stem cell differentiation to investigating how cancer cells manipulate gene expression, live-FISH opens a window into the dynamic world of gene regulation. It's a powerful tool, not just for observing life, but for understanding the very language that shapes it, gene by gene [55].

What sets the iChip apart is its ability to maintain bacteria in their "natural environment" by bringing that environment into the laboratory. The iChip can culture organisms from diverse sources such as soil, sea water, saliva, salt marsh, and wastewater bioreactors [97]. The mechanism involves a specialized "chip" comprising a rigid plastic structure housing 192 miniature wells. To employ the device, users simply immerse it in a bacterial sample mixed with agar, effectively capturing a single cell in each well. After the bacterial sample is collected, diffusion membranes are affixed to both sides of the chip, immobilizing the microbes. These membranes are then secured with plastic plates, and the iChip is introduced into a larger sample of its original environment, allowing nutrients to permeate through the membranes. Remarkably, this method has demonstrated a remarkable 30,000%increase in bacterial growth compared to standard agar plates [56].

## Exploration of Phylogenetic Diversity

Over the recent decades, a compelling realization has emerged, underscoring that the majority of microbial diversity existing on Earth remains largely uncharted within laboratory cultures. This vast realm of unknown microorganisms, often referred to as "MDM," asserts its numerical dominance across diverse major environments on our planet. Intriguingly, with the notable exception of the human body, where a substantial portion of microbes has been successfully cultured, these enigmatic microbial entities defy our conventional characterization methods. Our estimations indicate that approximately one-quarter of the global microbial cell population falls within phyla devoid of cultured relatives [57]. This revelation not only emphasizes the pervasive prevalence of these hitherto unstudied organisms but also raises intriguing implications for ecosystem functions. The substantial representation of uncultured phyla within Earth's microbial communities suggests a profound importance, indicating that unlocking the mysteries held by these never-before-explored microorganisms could yield crucial insights into the intricate dynamics of ecosystem functioning. The concept of MDM, with its dominance in diverse environments, beckons exploration and promises to unveil novel dimensions of microbial life critical to our understanding of Earth's ecosystems.

Microbial phylogenetics stands at the forefront of understanding the intricate genetic relationships among diverse groups of microorganisms, unraveling the evolutionary tapestry that has shaped their existence. Unlike higher organisms where the study of relationships often involves physiological and comparative anatomical methods, microorganisms pose unique challenges due to their microscopic nature and lack of distinct structures [58].

One of the key advantages of comparative genomics in microbial phylogenetics is its ability to overcome the limitations associated with traditional morphological and physiological approaches [59]. Microorganisms often lack discernible morphological features, and their physiological characteristics may be too subtle or dynamic to serve as reliable indicators of evolutionary relatedness. In contrast, the information encoded within the DNA, including gene sequences and genomic structures, serves as a stable and highly informative molecular record of evolutionary history [60]. Furthermore, advancements in DNA sequencing technologies have accelerated the pace of microbial phylogenetic research. High-throughput sequencing allows scientists to rapidly obtain vast amounts of genomic data from diverse microbial communities, facilitating more robust comparative analyses [61]. The integration of computational tools and bioinformatics in comparative genomics has also empowered researchers to handle large datasets, extract meaningful patterns, and construct more accurate phylogenetic trees. Generally, microbial phylogenetics, propelled by comparative genomics, enables biologists to navigate the intricate web of genetic relationships among microorganisms. This approach has become indispensable for tracing the evolutionary trajectories of microbial life forms, shedding light on their shared ancestry, adaptive strategies, and the mechanisms that have shaped the astounding diversity within the microbial world [62].

The vast majority of these organisms have not been found in pure culture, and we have only recently learned of their existence through shotgun sequencing (metagenomics) or cultivation-independent molecular surveys based on conserved marker genes, primarily small subunit ribosomal RNA (SSU rRNA) [50, 63]. Diversity estimates for Bacteria and Archaea are rising as more and more environments are thoroughly sequenced utilizing next-generation technologies. It is anticipated that there will be millions of different microbial "species" in existence [64]. These belong to at least 60 major lines of descent (phyla or divisions) within the bacterial and archaeal domains, according to SSU rRNA-based phylogeny, of which half lack cultivated representatives (so-called "candidate" phyla) [65]. Developments in the study of MDM could change our understanding of fundamental evolutionary concepts, including the ongoing controversy around the phylogenetic position of the Asgard archaea and the notion that the eukaryotic cell originated within the archaeal domain [66, 67]. According to estimates, "genomic dark matter" accounts for at least 80% of ambient genomic material [57, 68, 69], with the majority of this content being found in subsurface settings [70, 71].

Researchers commonly employ phylogenetic-driven techniques and canonical molecular markers for the targeted community profiling of MDM. Despite the utility of these approaches, the coding potential of these bacterial communities often proves challenging to access [72]. The retrieval of genomic information from these microorganisms faces limitations imposed by gene annotation methods that heavily depend on sequence similarity to proteins characterized from microbial cultures. Consequently, studying the FDM and pinpointing unique functions specific to uncultured organisms becomes a challenging task within this framework [73].

## New Bioinformatic and Functional Assays to Understand the Metabolic and Biosynthetic Capabilities of Uncultured Microbes

### Bioinformatics Tools for Analyzing Metagenomic and Single-Cell Genomic Data

In recent years, advancements in sequencing technologies and bioinformatics have led to the widespread use of high throughput sequencing for studying the composition, function, evolution, and interaction of microorganisms in different environments. This has significantly advanced the field of microbial ecology and resulted in numerous valuable scientific discoveries, particularly in relation to the gut microbiota and its impact on human health [74].

In contemporary microbiome research, diverse methodologies are employed to unveil nuanced insights at various levels of microbial information. These encompass the analysis of 16S rRNA, whole-genome shotgun (WGS; metagenome), and whole-transcriptome shotgun (metatranscriptome). 16S rRNA analysis relies on the conserved nature of the 16S rRNA gene to identify microbial entities. WGS analysis, on the other hand, harnesses information from all genes to decipher microbial identities at the species or even strain level, offering a granular understanding of microbial composition. The whole-transcriptome shotgun analysis provides a dynamic view of gene expression patterns, shedding light on the functionality of microbial communities [98]. Furthermore, whole-metabolite analysis furnishes a comprehensive inventory of chemicals within the target environment, facilitating the correlation of microbial abundance with downstream chemical dynamics. This multifaceted approach, incorporating distinct analytical techniques, empowers researchers to unravel the intricate tapestry of microbial ecosystems, elucidating not only their taxonomic composition but also shedding light on their functional roles and interactions within complex environments [75].

### Functional Assays to Decipher Metabolic and Biosynthetic Capabilities of Uncultured Microbes

For an extended period, the exploration of NPs relied on culture-dependent methods to isolate bioactive compounds. However, due to the rising issue of redundant isolation, these approaches have become less efficient for drug discovery over time [76]. Laboratory culture using nutrient-rich media can readily capture less than 1% of soil microorganisms, and the recovery percentage varies across environments, with higher counts in eutrophic settings like gut microbiomes. Innovative culture methods utilizing lower nutrient levels and extracts from natural environments prove effective in obtaining cultures from underrepresented bacterial phyla, especially when employing solid media [77]. Evidently, the advent of in situ incubation culturing methods has further diversified bacterial cultivation, enabling the discovery of diverse bacteria, some producing novel SMs like teixobactin, capable of inhibiting multidrug-resistant pathogens' growth [78]. While cultivation methods increasingly provide access to a broader spectrum of microbial genomes, metabolisms, and metabolites, their ability to sample environmental microbial diversity is inherently limited. A significant portion of microorganisms cannot be maintained in culture [79], and these methods, although yielding novel phylogenetic and chemical diversity, are time-consuming and necessitate focused attention on specific strains for optimal natural product discovery. Despite these limitations, these approaches persist in delivering unique contributions to both phylogenetic and chemical diversity, acting in tandem with culture-independent methods.

The anticipated metabolic substrates and resulting products, combined with environmental metadata, can be used to develop specific growth mediums. These can then be tested using a variety of cutting-edge cultivation methods, including microfluidics, cultivation chips, single-cell manipulation, and high-throughput cultivation. Additionally, techniques such as fluorescent in situ hybridization (FISH) and qPCR screening, based on the 16S rRNA gene, allow for the tracking and quantification of target organisms throughout the sampling, enrichment, cultivation, and recovery processes [99]. By leveraging these advanced cultivation approaches, we expect that a wide array of new target species will be successfully cultured. As metabolic reconstructions are typically based on homology, traditional approaches struggle to identify distinctive metabolic features or pathways in microorganisms that are phylogenetically or functionally different from well-characterized ones. The integration of -omics strategies, as previously outlined, offers a promising solution in this regard. By combining diverse layers of information, these strategies can help identify and address metabolic gaps that may have been overlooked in reconstructions based solely on sequence annotations. It is worth noting that software designed to leverage -omics data in constraints-based modeling predominantly focuses on integrating transcriptomics (expression) data [80]. In the future, greater emphasis should be placed on incorporating additional -omics datasets (such as proteomics, metabolomics, etc.) when modeling microbial metabolism. However, experiments involving detailed observations (*e.g.*, single knock-out mutants) are typically essential in providing a definitive solution to knowledge gaps in homology-based reconstructed metabolic models.

## Future Directions

The study of MDM has revealed a vast array of new biodiversity and chemo-diversity among uncultured microbes, opening up an exciting new frontier for natural product discovery. However, fully unlocking the potential of these microbial resources remains a significant challenge. To access a wider range of uncultured microbes, innovative approaches for long-term cultivation and simulating natural microbial environments are essential. Techniques like microfluidics, which enable precise control over small fluid volumes, can create microenvironments that closely replicate natural conditions [81]. The use of diffusible signaling factors to promote interspecies communication may enhance growth and metabolic activity, while co-culture systems—where multiple microbial species are grown together—can more accurately mimic the complexity of their native habitats [82]. Obviously, advances in single-cell genomics and transcriptomics also promise to revolutionize our understanding of MDM. These techniques can reveal biosynthetic pathways and regulatory mechanisms, providing insights into the metabolic potential of uncultured microbes without requiring cultivation. By employing these emerging methods, researchers may access a much larger portion of MDM, potentially uncovering vast libraries of NPs that remain undiscovered [83].

To achieve meaningful progress in discovering novel NPs from MDM, large-scale collaborative efforts will likely be necessary. Such consortia should combine cultivation experiments, genomic analyses, and high-throughput screening methods. By pooling resources and expertise, the scientific community will be better positioned to address the complexities of MDM, paving the way for transformative discoveries in the years ahead. As our techniques continue to evolve and our understanding of microbial ecosystems deepens, the potential to discover new NPs with applications in medicine, agriculture, and biotechnology will grow even stronger.

## Conclusion

The rapid development of DNA sequencing technology has revealed the vast diversity of microorganisms in nature that have not yet been cultured. The challenge now is to use these data to support cultivation and investigate ecological questions about the role of microorganisms and the microbiome in their natural habitats. Metagenomic sequencing has greater potential than providing new insights into microbiome function, as it also opens new opportunities for isolating and culturing microorganisms. In particular, long-read DNA sequencing technologies, greater sequencing depth, and advanced bioinformatics methods can improve the quality of genomic data for individual organisms and species on MAG and enable more accurate metabolic interpretation. These data support the development of novel media, genome-based antibody engineering, and targeted gene cultures that have led to the successful isolation of diverse microorganisms from diverse environments. In fact, Opportunities for using metagenomic and culture-independent sequence data for microbial isolation are numerous and can significantly increase the chance of successfully culturing the target organism of interest. However, it is recognized that the process of directed culture is complex and currently requires a good understanding of genomic data to predict culture needs or advanced technologies that are not readily available to all microbiology laboratories. However, this targeted approach can shorten the overall time to isolate a specific target microorganism and introduce it into culture. The challenge now is to exploit the rich culture-independent genetic data for targeted high-throughput culture, which, combined with advances in culture methods, may lead to new breakthroughs in capturing the uncultivated majority of the population.

## Figures and Tables

**Fig. 2 F2:**
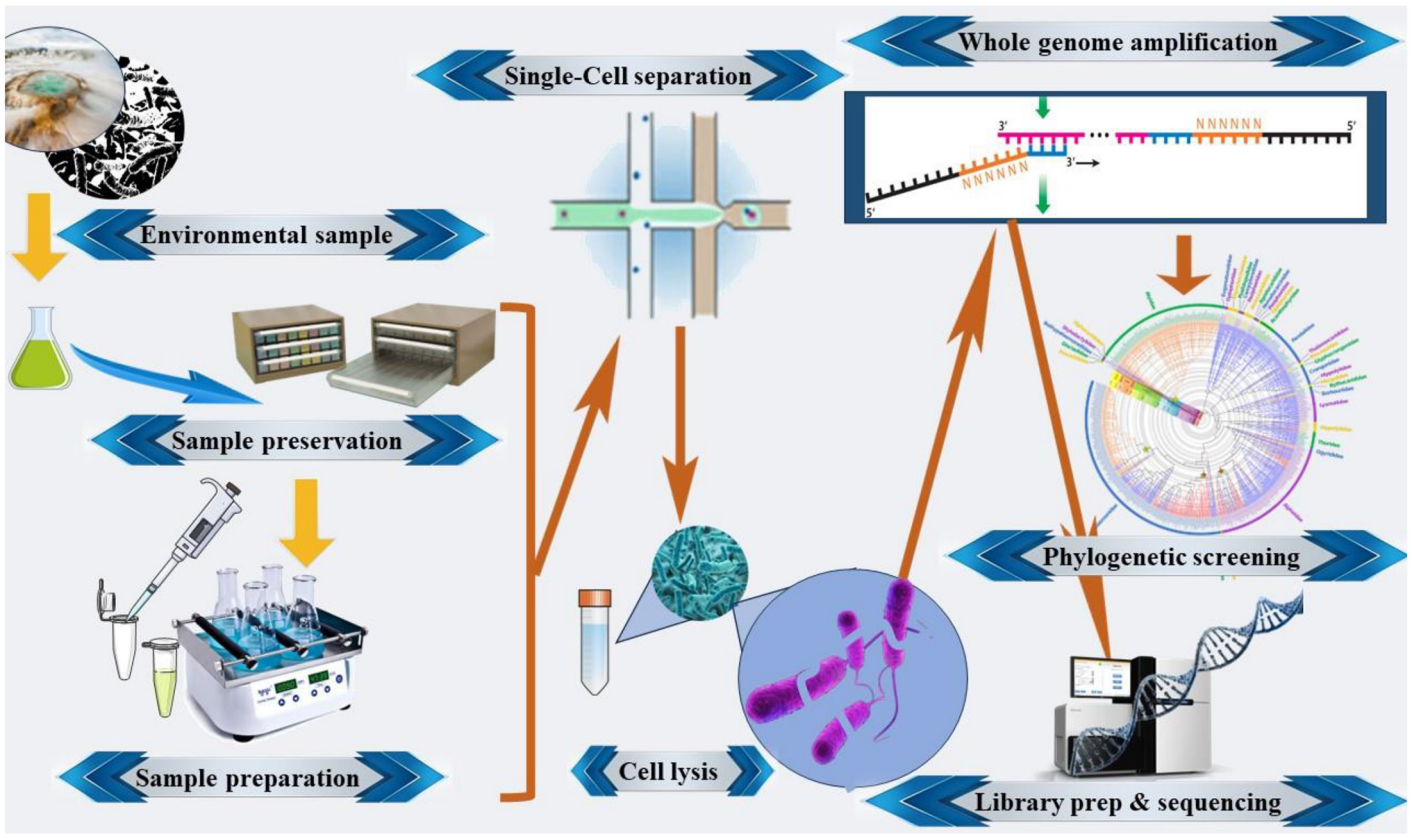
The single-cell genomics workflow encompasses a series of meticulously orchestrated steps.
